# Multi-Marker Strategy in Heart Failure: Combination of ST2 and CRP Predicts Poor Outcome

**DOI:** 10.1371/journal.pone.0157159

**Published:** 2016-06-16

**Authors:** Anne Marie Dupuy, Corentin Curinier, Nils Kuster, Fabien Huet, Florence Leclercq, Jean Marc Davy, Jean Paul Cristol, François Roubille

**Affiliations:** 1 Department of Biochemistry, Centre Ressources Biologiques de Montpellier, University Hospital of Montpellier, Montpellier, France; 2 Cardiology Department, University Hospital of Montpellier, Montpellier, France; 3 PhyMedExp, University of Montpellier, INSERM U1046, CNRS UMR 9214, Montpellier, France; Boston University, UNITED STATES

## Abstract

Natriuretic peptides (BNP and NT-proBNP) are recognized as gold-standard predictive markers in Heart Failure (HF). However, currently ST2 (member of the interleukin 1 receptor family) has emerged as marker of inflammation, fibrosis and cardiac stress. We evaluated ST2 and CRP as prognostic markers in 178 patients with chronic heart failure in comparison with other classical markers such as clinical established parameters but also biological markers: NT-proBNP, hs-cTnT alone or in combination. In multivariate analysis, subsequent addition of ST2 led to age, CRP and ST2 as the only remaining predictors of all-cause mortality (HR 1.03, HR 1.61 and HR 2.75, respectively) as well as of cardiovascular mortality (HR 1.00, HR 2.27 and HR 3.78, respectively). The combined increase of ST2 and CRP was significant for predicting worsened outcomes leading to identify a high risk subgroup that individual assessment of either marker. The same analysis was performed with ST2 in combination with Barcelona score. Overall, our findings extend previous data demonstrating that ST2 in combination with CRP as a valuable tool for identifying patients at risk of death.

## Introduction

Heart failure (HF) results from multiple conditions leading to structural and functional changes. Indeed, HF is not simply a mechanical failure of the heart pump. In addition to the classical sympathetic overstimulation, various pathophysiological ways are involved. First, one of the main pathophysiological ways leading to HF is myocardial stress resulting in neurohormonal activation by natriuretic peptides, including B-type natriuretic peptide (BNP) and its amino-terminal cleavage fragment, NT-proBNP. Their interest is well established in both diagnosis and prognosis [1]. Natriuretic peptides are recommended by 2013 ACC/AHA guidelines [2] and 2012 ESC guidelines for diagnosis and prognosis in chronic HF (class I), and for guidance of evidence based treatments (2013 ACC/AHA guidelines [2], class IIa). Secondly, local and systemic inflammation are clearly involved and reflected in clinical practice mainly by C-reactive protein (CRP) which is correlated with the severity and prognosis of HF [3–7]. Third, ventricular remodelling is also involved in association with hypertrophy and myocyte death and excessive renewal of the extracellular matrix [8]. Fourth, iterative myocardial injuries could participate and could be reflected by low-level increased troponin without any clinically significant ischemic events. Consistently, low-level elevation of troponins were found correlated with prognosis [9]. Finally, other markers such as oxidative stress or kidney dysfunction have been shown to be involved in the onset and development of HF [4].

Currently, natriuretic peptides (BNP and NT-proBNP) are recognized as gold-standard predictive markers in HF. However, when considered alone natriuretic peptides are not tailored to reflect the various pathophysiologic pathways in HF. Other markers might be then useful to improve risk stratification for patients with HF. Among emerging markers integrating inflammation, fibrosis and cardiac stress [10], ST2 (member of the interleukin 1 receptor family), has emerged as a promising prognostic marker. Recently, FDA has recognized the increasing importance of ST2 in chronic HF. ST2 is included in a novel bio-clinical algorithm (Barcelona bio-heart failure risk calculator) in association with NT-proBNP and high-sensitivity cardiac troponin T (hs-cTnT), which allowed accurately prediction of death at 1, 2, and 3 years [11].

In this context, the aim of our study was to evaluate ST2 as prognosis marker in a population with chronic HF in comparison with other classical markers such as clinical established parameters but also biological markers: NT-proBNP, CRP, hs-cTnT alone or in combination.

## Methods

### Study population

Between May 2010 and February 2011, 182 patients with stable HF were prospectively included in a single University Hospital (CHRU Montpellier, France). All participants provided written informed consent. The protocol was performed according to the principles of the Declaration of Helsinki, approved by the Ethic Committee of Montpellier and the biological collection registered by the French government (research Ministery, # DC-2009-1052).

To be eligible to the study, the patients were previously (at least 6 months before the inclusion) diagnosed with acute or chronic HF, as recommended by the European Society of Cardiology [12]. Main inclusion criteria were the ability to give informed consent, age>18 years and confirmed diagnosis of HF, irrespectively of the cause or treatments. All clinical available data at the time of initial visit were collected by two cardiologists from the medical records of each patient. Comorbidities such as hypertension, diabetes, chronic obstructive pulmonary disease (COPD), chronic kidney disease, pulmonary embolism, myocarditis, smoking habit, dyslipidemia were recorded. Other clinical variables as age, gender, New York Heart Association (NYHA) class, ischemic etiology, left ventricular ejection fraction (LVEF), medications (angiotensin converting enzyme inhibitor: ACE or ARBs, betablockers, Ivabradine, aldosterone antagonists use, diuretics use, anti platelet agents and anticoagulants use, digoxin, statin, antiarrhythmic and others medications use) and laboratory values were also reported (Table 1). Main exclusion criteria were unstable angina or acute coronary syndrome in the past month, cardiac surgery and chemotherapy.

**Table 1:** Baseline characteristics of all patients with chronic HF according to all cause of mortality.

	Study Population n = 178	Alive, n = 112	Deceased, n = 66	p
Demographic Characteristics				
Age, years	75.43 (66.4–81.2)	72.3 (63.6–78.5)	79.19 (72.5–83.7)	**0.001**
Gender, n (%)				-
F	56 (31.5)	38 (33.9)	18 (27.3)	
M	122 (68.5)	74 (66.1)	48 (72.7)	-
Death from cardiovascular cause, n (%)	36 (20.2)	0 (0)	36 (54.5)	<0,001
Co-morbidities, n (%)				
Hypertension	113 (63.5)	64 (57.1)	49 (74.2)	**0.02**
Diabetes	63 (35.4)	30 (26.8)	33 (50)	**0.002**
COPD	39 (21.9)	23 (20.5)	16 (24.2)	0.558
Chronic kidney disease	38 (21.3)	16 (14.3)	22 (33.3)	**0.004**
Pulmonary embolism	11 (6.2)	6 (5.4)	5 (7.6)	0.746
Myocarditis	1 (0.6)	1 (0.9)	0 (0)	1
Smoking habit	84 (47.2)	56 (50)	28 (42.4)	0.369
Dyslipidemia	84 (47.2)	52 (46.4)	32 (48.5)	0.863
Heart Failure Characteristics, n (%)				
NYHA class				
I	10 (5.6)	9 (8.1)	1 (1.5)	
II	54 (30.5)	39 (35.1)	15 (22.7)	
III	82 (46.3)	49 (44.1)	33 (50)	
**IV**	31 (17.5)	14 (12.6)	17 (25.8)	**0.023**
Ischemic cardiopathy	86 (53.1)	53 (51)	33 (56.9)	0.504
Defibrillator	51 (28.7)	34 (30.4)	17 (25.8)	0.621
Medication Use, n (%)				
ACE inhibitors or ARBs	123 (69.1)	86 (76.8)	37 (56.1)	**0.006**
Betablockers	48 (27)	28 (25)	20 (30.3)	0.468
Ivabradine	165 (92.7)	107 (95.5)	58 (87.9)	0.077
Aldosterone antagonists	54 (30.3)	44 (39.3)	10 (15.2)	**0.001**
Diuretics	127 (71.3)	78 (69.6)	49 (74.2)	0.631
Anti platelet agent	14 (7.9)	9 (8)	5 (7.6)	1
Anticoagulant therapy	18 (10.1)	9 (8)	9 (13.6)	0.304
Digoxin	8 (4.5)	2 (1.8)	6 (9.1)	**0.034**
Statin	16 (9)	9 (8)	7 (10.6)	0.589
Anti-arrhythmic	11 (6.2)	7 (6.2)	4 (6.1)	1
Others	8 (4.5)	4 (3.6)	4 (6.1)	0.478
Clinical Measures				
Body Mass Index, kg/m²	26.1 (22.9–29.8)	26.2 (22.6–30.4)	25.7 (23.1–29.3)	0.755
LVEF, %	35 (25–45)	35 (27–45)	35 (25–45)	0.296
Biomarkers				
Urea, mmol/L	9.45 (6.8–14.1)	8.4 (6.2–12.0)	11.7 (8.4–17.8)	**<0.001**
Sodium, mmol/L	138 (135.0–140)	138 (135.7–140)	137.5 (135–140)	0.382
Creatinine, μmol/L	101 (82.2–136.5)	91 (78–124.5)	116.5 (92.2–145.2)	**0.001**
eGFR CKD-EPI, mL/min/1,73m²	55.83 (38.4–76.9)	62.14 (44.7–83.7)	49.41 (31.4–66.2)	**0.001**
NT-proBNP, pg/mL	2344 (853–5616)	1797 (574–3492)	3761.5 (1686–10679)	**<0.001**
Hs-cTnT, ng/L	43.13 (19.9–127.9)	33.55 (15.8–87.7)	56.79 (30.9–152.4)	**0.002**
CRP, mg/L	6.05 (2.4–25.5)	4.05 (2–16.5)	14.9 (2.8–32.8)	**0.004**
ST2, ng/mL	37.4 (19.5–69.7)	28.22 (16.4–50.6)	52.4 (30.8–106.7)	**<0.001**

Data presented as median (1st quartile–3rd quartile), and patient number with percent of total.

COPD: Chronic obstructive pulmonary disease, NYHA: New York Heart Association, ACE: Angiotensin Converting Enzyme, ARB: *Angiotensin Receptor Blocker*, *LVEF*: *left ventricular ejection fraction*, eGFR CKD-EPI: estimated glomerular filtration rate Chronic Kidney Disease—Epidemiology Collaboration.

### Follow up and outcomes

After inclusion, patients were followed by their cardiologist who decided the monitoring frequency according to guidelines. In December 2014, a dedicated physician was in charge of collecting the clinical data: primary endpoint (deaths of all causes), as well as the secondary endpoints (cardiovascular deaths, HF), and prescription of drugs including beta-blockers, ACE inhibitors or ARB, statins and the dosage of loop diuretics (mg/d).

Data collection was performed by analyzing the medical files and by phone with the general practitioner, the patient or the family. Cardiovascular death included death resulting from an acute myocardial infarction, sudden cardiac death, death due to HF, death due to stroke, death due to cardiovascular procedures, death due to cardiovascular hemorrhage.

### Biochemical analysis

Venous blood was collected in dry and EDTA tubes and was immediately centrifuged (the samples are transported in a mean total delay of less than 3 hours (all inclusive until frozen); in the biochemistry lab 95% are treated in less than an hour and a half and 50% in less than one hour) and frozen (-80°C) until tested four years later.

From dry tube, the NT-proBNP and hs-cTnT levels were determined using an immuno-electrochemiluminescence assay on the Cobas8000/e602® immunochemistry system (Roche Diagnostics, Meylan, France). Determination of CRP was run on the Cobas8000/e502® analyzer (Roche Diagnostic, Meylan, France) using immunoturbidimetric method.

We used 1 aliquot of the EDTA plasma aliquot for the determination of ST2 (this was the first thawing.), and all patients samples were measured in 1 batch 4 years after the recruitment period. ST2 plasma concentrations were measured with a high sensitivity sandwich monoclonal immunoassay (Presage© ST2 assay, Critical Diagnostics, San Diego, California distributed in France by Eurobio society).

All other biochemistry parameters as urea, creatinine, sodium were performed on Cobas 8000/c701® and ISE (Roche, Meylan, France).

### Statistical analysis

Categorical data are expressed as count (percentage). Continuous data are expressed as mean (standard deviation) or median (1st quartile–3rd quartile) for normal and skewed distributions, respectively. Comparison between two groups was performed using Mann-Whitney U test. Correlation coefficients reported were based on a non-parametric method (Spearman rank).

Survival curves were generated using Kaplan-Meier non parametric estimator. Log-rank test was used to compare multiple survival distributions. Survival analysis was performed using Cox proportional hazard model. A multivariate baseline model was fitted including variables potentially associated with mortality in HF patients. Baseline regression model included age, gender, vascular risk factor, pulmonary risk factor, dyslipidemia, NYHA class, ischemic cardiopathy, LVEF, biological biomarkers including sodium, NT-proBNP and hs-cTnT. The vascular risk factor variable included diabetes, hypertension and estimated glomerular filtration rate (eGFR CKD-EPI equation). The pulmonary risk factor variable combined COPD and tobacco habit. The model was then augmented with CRP and ST2 levels to test whether these biomarkers have an incremental value for predicting mortality. Because of skewed distributions, biomarkers concentrations were log-transformed before modeling. Harrell’s c (C-statistic) was used for evaluating the discrimination ability of the model. Goodness of fit was assessed using Akaike’s Information Criterion (AIC). Comparison of two nested models was performed using the likelihood ratio test.

The Barcelona bio-heart failure risk calculator (BCN Bio-HF calculator) is an estimator of the risk of death in patients with HF described by Lupon et al. [11] The BCN Bio-HF calculator is based on eight independent models, depending on available data. The clinical models account of clinical and biological characteristics and treatments to predict the risk of mortality at 1, 2 and 3 years. When available, the clinical + biomarker models can also take account of biomarkers concentrations (hs-cTnT, NT-proBNP, ST2) to refine predictions Using these models, prognostic index were computed for each patient in our population. In order to estimate risk stratification allowed by clinical and clinical + biomarkers indexes, Cox models were fitted for prediction of all-cause and cardiovascular mortality. To simplify reading BCN Bio-HF calculator was called Barcelona score or BCN.

The clinical benefit in risk prediction of adding a biomarker to the clinical model was further assessed by reclassification analysis, including both the net reclassification improvement (NRI) and the integrated discrimination index (IDI) [13–14]. Reclassification analysis was proposed to evaluate added usefulness of a new biomarker over preexisting models. Continous NRI as proposed by Pencina et al. [15] is an extension of NRI, applicable to survival data and doesn’t need to define risk categories, which are known to influence NRI. Statistical analysis was performed using R 3.1.3 (R Development Core Team, Vienna, Austria). A two-sided p<0.05 was considered significant.

## Results

### Performances of ST2 as discriminator of patient risk

**Fig 1:**
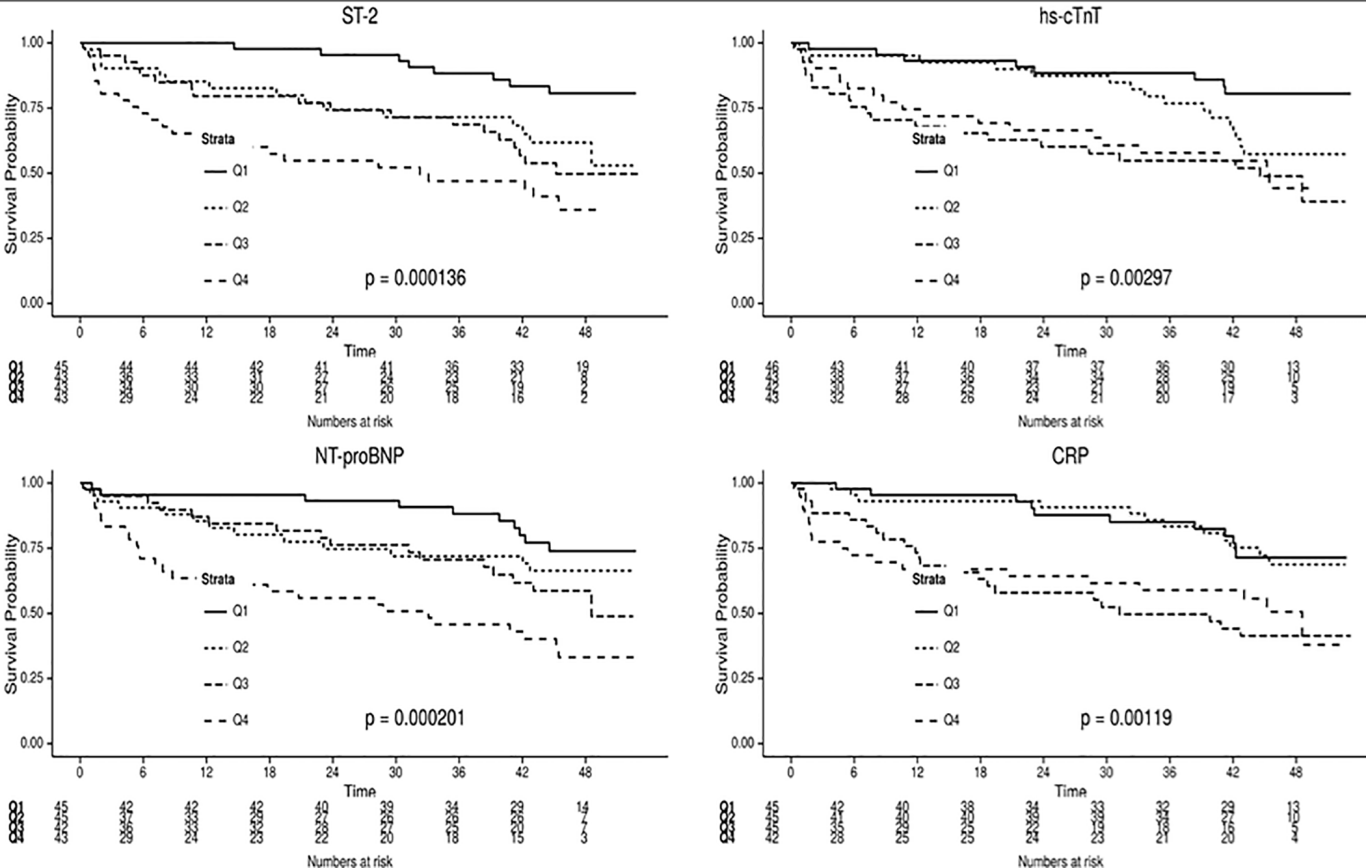
Kaplan Meier curves for all-cause mortality based on quartiles of ST2, hs-cTnT, NT-proBNP and CRP.

Out of 182 consecutive patients included from May 2010 to February 2011, biochemical measurements and vital status were available for 178, which were included in our analysis. Over a median follow-up period of 42.3 months (range 12.3 to 47.1 months) there were 66 deaths. Clinical and biochemical variables in survivors vs deceased are reported in Table 1. Of the 66 patients who died during the 4 years of follow-up, 36 (54.5%) died from cardiovascular causes and 30 (45.5%) from other causes. Among all comorbidities, only hypertension, diabetes and chronic kidney disease were associated with excess mortality. The NYHA class at baseline tended to be higher among decedents than survivors. Median LVEF was not significantly different between the 2 groups of patients. All median biochemical parameters values including ST2 biomarker were statistically significantly higher in deceased patients versus alive. Mortality clearly increased across quartiles of ST2, NT-proBNP, CRP and hs-cTnT (Fig 1, p<0.001 for all).

In univariate Cox regression analysis over the 42 months, age, vascular risk factor, NYHA class, and the four biomarkers (NT-proBNP, hs-cTnT, CRP and ST2) were associated with all cause as well as cardiovascular mortality (Tables 2 and 3, respectively). In the multivariate baseline model, age, and NT-proBNP, remained independent predictors of all cause mortality, alone the NT-proBNP of cardiovascular mortality (Tables 2 and 3, respectively). After addition of CRP to this model, NYHA was no more associated with mortality whereas age, NT-proBNP and CRP remained significant predictors. Subsequent addition of ST2, led to age, CRP, and ST2 as the only remaining predictors of all-cause mortality (HR 1.039, 95%CI 1.01–1.068, HR 1.61, 95%CI 1.03–2.52 and HR 2.75, 95%CI 1.20–6.28, respectively) as well as of cardiovascular mortality (HR 1.04, 95%CI 1.00–1.089, HR 2.27, 95%CI 1.19–4.33 and HR 3.78, 95%CI 1.18–12.15, respectively). Only ST2 provided incremental value associated with a greatest and significant increase of the c-statistic from 0.647 (in the model that included only age and sex) to 0.717 (p<0.001) (model including age, gender and ST2).

**Table 2:** Predictors of all-cause mortality in the study population of HF.

		Univariate model		Baseline model		Baseline model + CRP		Baseline model+ CRP + ST2	
Variable		HR [95% CI]	p	HR [95% CI]	p	HR [95% CI]	p	HR [95% CI]	p
Age		1.048 [1.021–1.075]	**< 0.001**	1.03 [1.002–1.058]	**0.037**	1.03 [1.002–1.058]	**0.032**	1.039 [1.01–1.068]	**0.008**
Gender	M	1.209 [0.7–2.089]	0.496	1.341 [0.715–2.516]	0.361	1.44 [0.762–2.722]	0.261	1.391 [0.738–2.624]	0.308
Vascular risk factor		4.325 [1.356–13.798]	0.013	2.53 [0.736–8.695]	0.14	3.111 [0.893–10.836]	0.075	3.185 [0.905–11.211]	0.071
Pulmonary risk factor		0.951 [0.58–1.558]	0.841	1.199 [0.704–2.043]	0.504	1.371 [0.793–2.368]	0.259	1.419 [0.815–2.469]	0.216
Dyslipidemia		1.129 [0.689–1.852]	0.63	0.982 [0.555–1.737]	0.951	0.748 [0.41–1.364]	0.344	0.824 [0.45–1.51]	0.531
NYHA class		1.728 [1.258–2.374]	**0.001**	1.411 [0.983–2.023]	0.062	1.292 [0.904–1.846]	0.16	1.126 [0.763–1.661]	0.549
Ischemic cardiopathy		1.056 [0.644–1.731]	0.83	0.884 [0.494–1.581]	0.677	0.905 [0.508–1.612]	0.735	0.883 [0.495–1.575]	0.674
LVEF		0.989 [0.97–1.008]	0.247	0.999 [0.974–1.024]	0.919	0.998 [0.974–1.023]	0.886	0.994 [0.97–1.019]	0.652
Sodium		0.981 [0.923–1.042]	0.526	0.975 [0.914–1.042]	0.458	0.965 [0.905–1.03]	0.287	0.971 [0.912–1.034]	0.364
NT-proBNP (log 10)		3.046 [1.972–4.705]	**< 0.001**	2.654 [1.516–4.647]	**0.001**	2.453 [1.417–4.245]	**0.001**	1.762 [0.981–3.164]	0.058
Hs-cTnT (log 10)		1.669 [1.218–2.287]	**0.001**	0.865 [0.549–1.365]	0.534	0.793 [0.502–1.253]	0.32	0.742 [0.466–1.182]	0.208
CRP (log 10)		2.109 [1.44–3.089]	**< 0.001**			1.839 [1.179–2.867]	**0.007**	1.614 [1.031–2.527]	**0.036**
ST2 (log 10)		3.915 [2.31–6.635]	**< 0.001**				** **	2.751 [1.205–6.28]	**0.016**

CI: confidence interval; COPD: Chronic obstructive pulmonary disease, NYHA: New York Heart Association, *LVEF*: *left ventricular ejection fraction*, eGFR CKD-EPI: estimated glomerular filtration rate Chronic Kidney Disease—Epidemiology Collaboration.

**Table 3:** Predictors of cardiovascular mortality in the study population of HF.

		Univariate model		Baseline model		Baseline model + CRP		Baseline model+ CRP + ST2	
Variable		HR [95% CI]	p	HR [95% CI]	p	HR [95% CI]	p	HR [95% CI]	p
Age		1.057 [1.02–1.096]	**0.003**	1.032 [0.993–1.074]	0.113	1.033 [0.994–1.073]	0.096	1.046 [1.005–1.089]	**0.027**
Gender	M	1.009 [0.492–2.07]	0.98	0.992 [0.426–2.307]	0.984	1.095 [0.463–2.59]	0.836	1.037 [0.439–2.452]	0.934
Vascular risk factor		7.087 [0.969–51.85]	0.054	3.977 [0.492–32.167]	0.196	5.591 [0.674–46.377]	0.111	6.025 [0.72–50.437]	0.098
Pulmonary risk factor		1.03 [0.525–2.021]	0.931	1.482 [0.705–3.115]	0.299	1.91 [0.878–4.152]	0.103	1.994 [0.908–4.379]	0.086
Dyslipidemia		0.973 [0.494–1.915]	0.936	0.757 [0.342–1.674]	0.492	0.493 [0.213–1.139]	0.098	0.552 [0.241–1.269]	0.162
NYHA class		1.606 [1.047–2.464]	**0.03**	1.24 [0.752–2.044]	0.399	1.103 [0.676–1.798]	0.695	0.902 [0.521–1.56]	0.712
Ischemic cardiopathy		1.099 [0.561–2.155]	0.783	1.034 [0.464–2.305]	0.935	1.083 [0.491–2.387]	0.844	1.094 [0.496–2.411]	0.824
LVEF		0.988 [0.963–1.014]	0.37	0.994 [0.961–1.029]	0.748	0.993 [0.962–1.025]	0.669	0.987 [0.955–1.019]	0.414
Sodium		0.974 [0.898–1.056]	0.525	0.976 [0.894–1.066]	0.594	0.955 [0.874–1.043]	0.306	0.963 [0.886–1.047]	0.381
NT-proBNP (log 10)		3.949 [2.15–7.254]	**< 0.001**	3.505 [1.578–7.784]	**0.002**	3.114 [1.432–6.772]	**0.004**	1.968 [0.859–4.508]	0.109
Hs-cTnT (log 10)		1.865 [1.22–2.85]	**0.004**	0.874 [0.475–1.607]	0.664	0.739 [0.396–1.377]	0.34	0.674 [0.356–1.277]	0.226
CRP (log 10)		2.694 [1.587–4.573]	**< 0.001**			2.654 [1.385–5.089]	**0.003**	2.273 [1.191–4.336]	**0.013**
ST2 (log 10)		4.987 [2.469–10.072]	**< 0.001**					3.786 [1.18–12.15]	**0.025**

CI: confidence interval; COPD: Chronic obstructive pulmonary disease, NYHA: New York Heart Association, LVEF: left ventricular ejection fraction, eGFR CKD-EPI: estimated glomerular filtration rate Chronic Kidney Disease—Epidemiology Collaboration.

We applied the BCN Bio-HF calculator [10] to assess the predictive ability of mortality in our population. The model with the three biomarkers (ST2, NT-proBNP and hs-cTnT) had a c-statistic of 0.715 for all cause of mortality and 0.751 for predictive ability of cardiovascular mortality. With a c-index of 0.751, the model including biomarkers was relevant in our population.

### CRP enhances the predictive power of ST2 and Barcelona score

**Fig 2:**
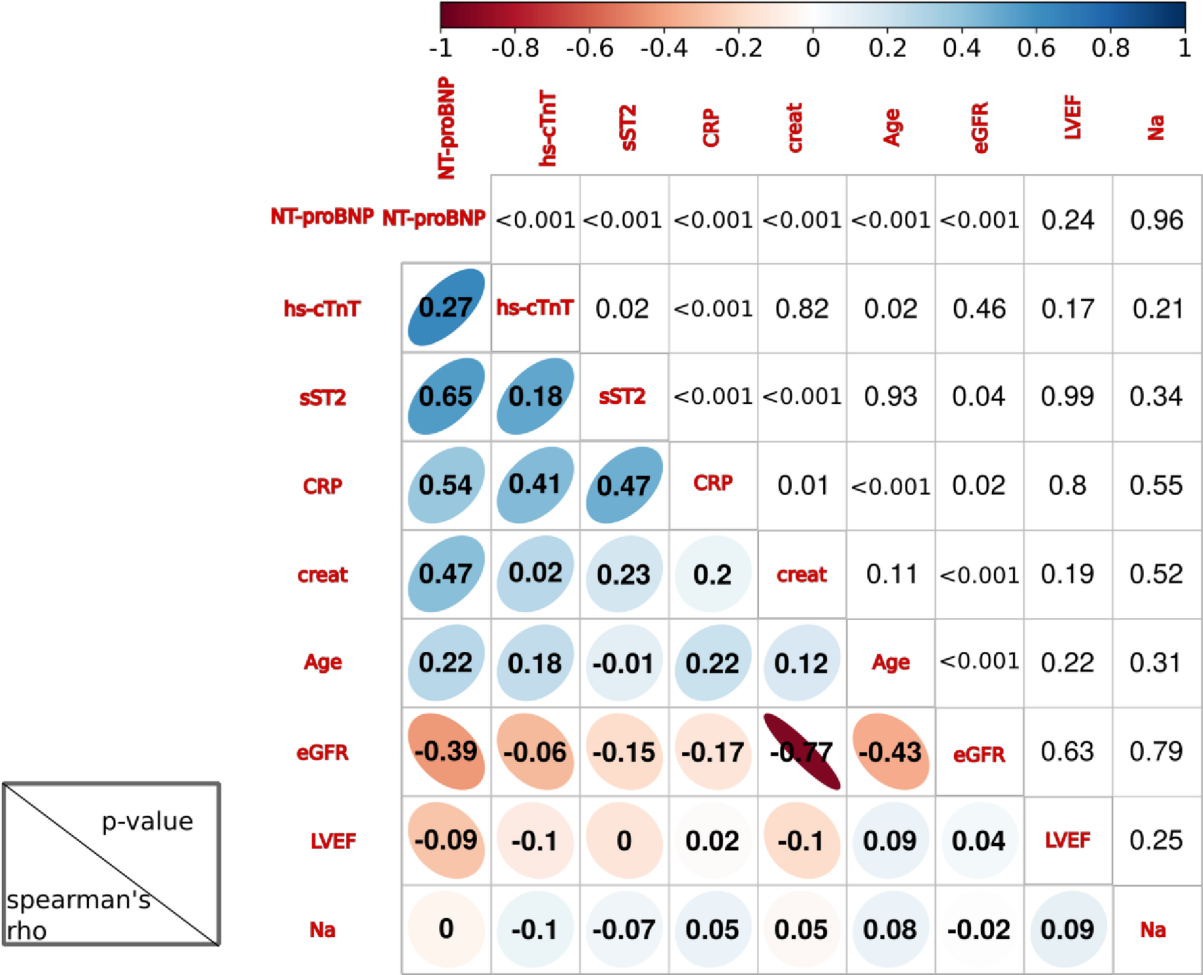
Correlation analysis between each biomarker included in this study.

First at all, we analysed the correlation of ST2 and CRP with all other cardiac biomarkers (NT-proBNP and hs-cTnT). Overall, the 4 biomarkers showed a significant correlation with each other (Fig 2).

**Fig 3:**
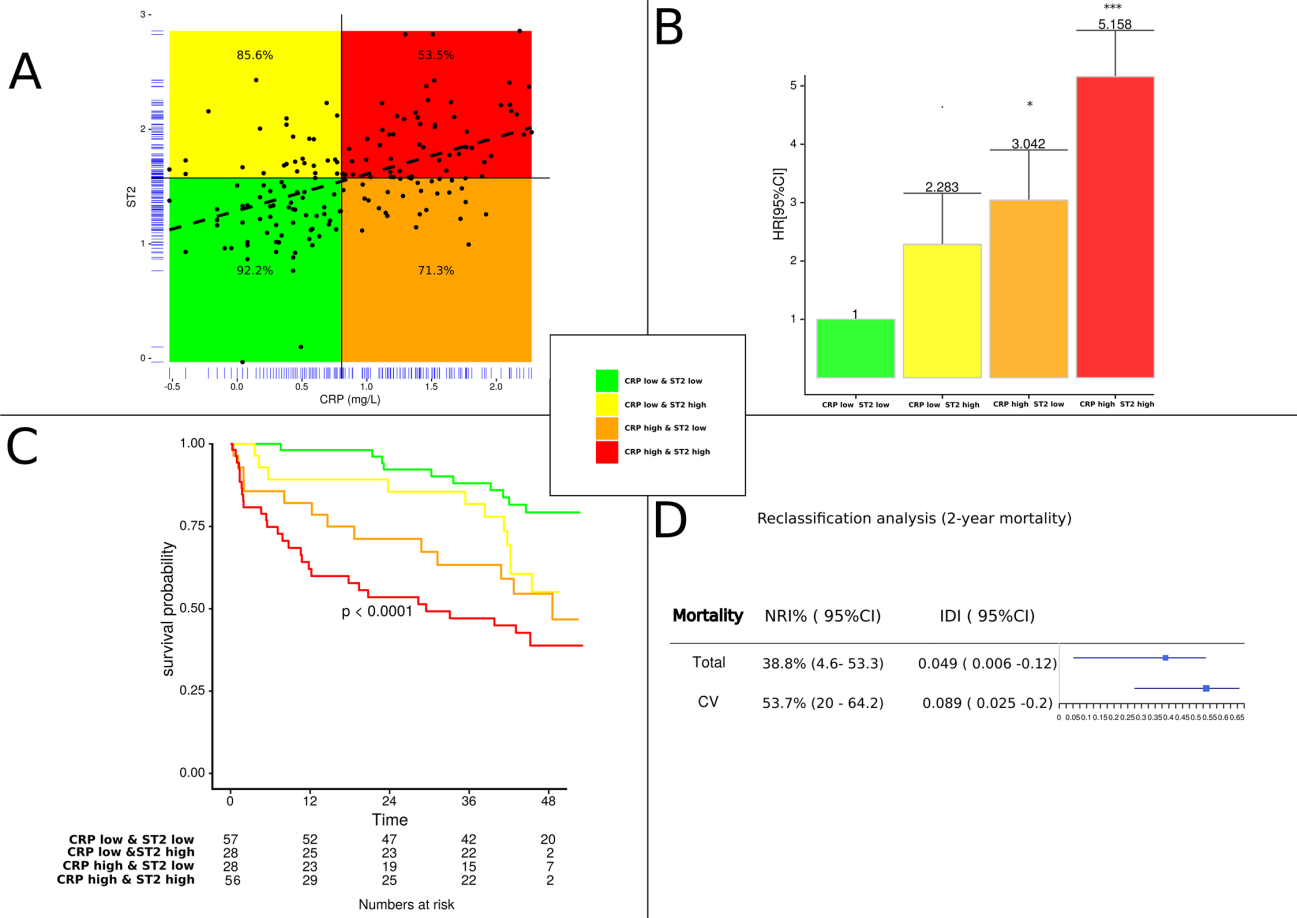
**Association of ST2 with CRP levels dichotomized as high or low according to the median of each biomarker (A) Correlation analysis with survival rate in %, (B) Adjusted hazard ratio of death according to combined criteria as median of ST2 and CRP and (C) Kaplan Meier curves for all-cause mortality according to combination of median of ST2 and CRP and (D) Reclassification of two years risk of death for all cause or cardiovascular (CV) mortality. NRI and IDI values were reported when CRP was added to the clinical model in combination with high level of ST2.** NRI: net reclassification index; IDI: integrated discrimination index.

To further evaluate the relationship between ST2 and CRP, Fig 3 shows the plotted correlation that we divided into four parts: patients with high (above median) and low (below median) ST2 and high (above median) and low (below median) CRP. The 2-year survival rates in the group of patients with high levels of ST2 alone and CRP alone were 85% and 71%, respectively. The presence of both elevated biomarkers was associated with a strikingly steeply rising incremental risk of death, reaching more 47% (Fig 3A). The additive value of ST2 and CRP was further analyzed by Cox proportional hazard model. After adjustment for age, gender, co-morbidities, NYHA class, LVEF, biological biomarkers the combined increase of ST2 and CRP was significant for predicting worsened outcomes (HR 5.158, CI 2.33–11.41, p = <0.0001, C-statistic = 0.745) (Fig 3B). Kaplan-Meier curves confirmed the threshold effect between each group for prediction of all cause of mortality. Patients with elevations in both ST2 and CRP had markedly increased, indicating that assessment of both ST2 and CRP was more effective at identifying a high risk subgroup that individual assessment of either marker (Fig 3C). Considering 2-year mortality, CRP improved individual risk prediction in reclassification analysis (Fig 3D). Risk classification analysis was significantly improved with the addition of CRP particularly for cardiovascular mortality (53.7% reclassified; p = 0.005). The improvement is less pronounced for all-cause mortality (38.8% reclassified; p = 0.035). IDI confirmed these results of the reclassification analysis for CRP.

**Fig 4:**
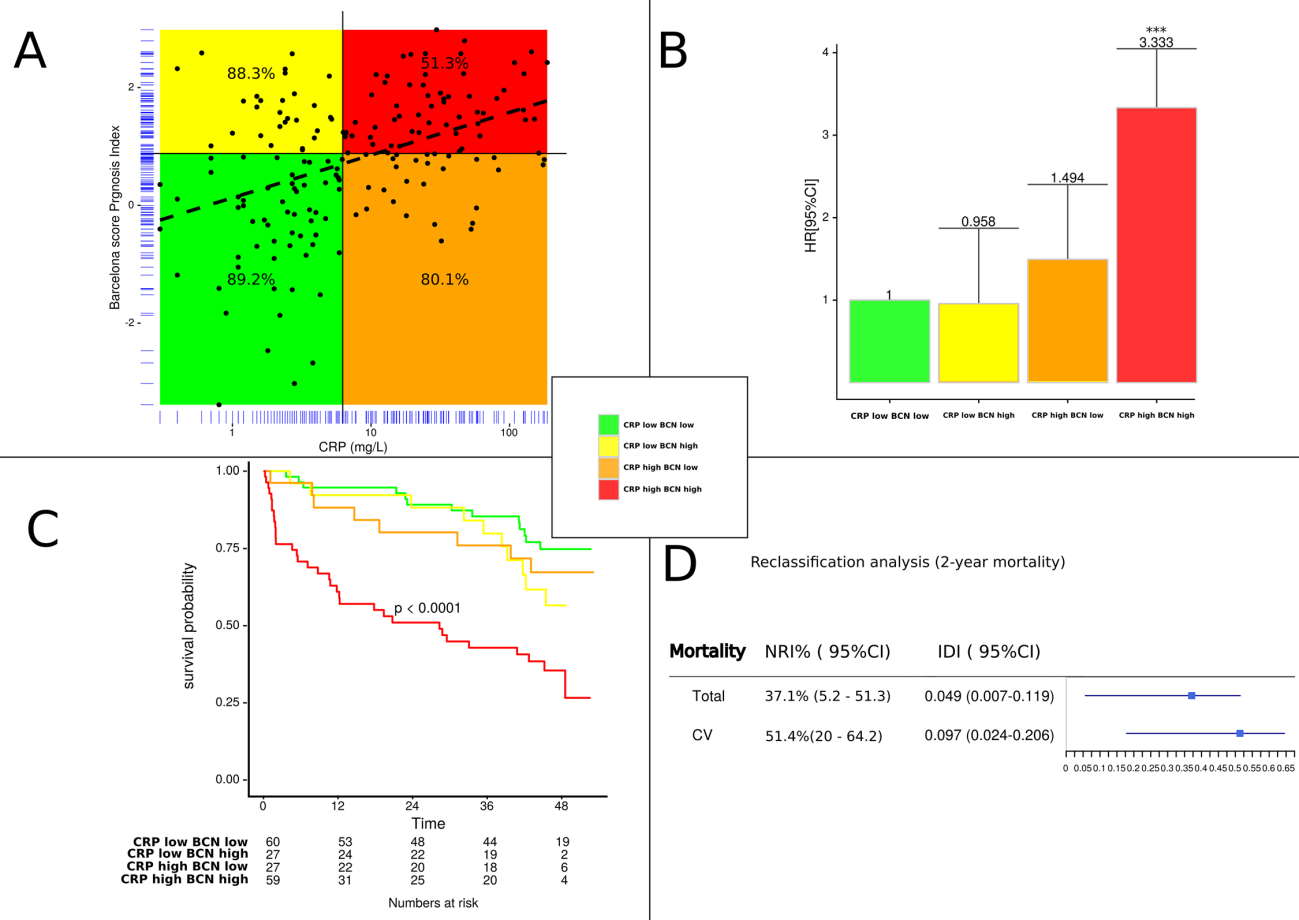
**Association of ST2 with Barcelona score levels dichotomized as high or low according to the median of each biomarker (A) Correlation analysis with survival rate, (B) Adjusted hazard ratio of death according to combined criteria as median of ST2 and Barcelona score (C) Kaplan Meier curves for all-cause mortality according to combination of median of ST2 and Barcelona score and (D) Reclassification of two years risk of death for all cause or cardiovascular (CV) mortality. NRI and IDI values were reported when CRP was added to the clinical model in combination with high BCN value.** NRI: net reclassification index; IDI: integrated discrimination index; BCN: Barcelona bio-heart failure risk calculator (BCN Bio-HF calculator).

The same analysis was performed with the combination of CRP and Barcelona score. The correlation analysis between Barcelona score and CRP showed that combined assessment of these two biomarkers added predictive power with close 51.3% 2-year survival rate vs 88% if we considered BCN alone or 80% with CRP alone (Fig 4A). Similarly, to the association ST2 and CRP, the combined increases of Barcelona score and CRP substantially augmented risk (HR 3.333, CI 1.629–6.817, p = 0.01) (Fig 4B). Kaplan Meier analyses also showed significant separation of survival curves for patients with low and/or high levels of BCN Bio-HF score and CRP for 4-year survival. Patients with both BCN Bio-HF score and CRP above their median experienced a mortality rate of around 65%, equaling the mortality rate for patients with high CRP and BCN levels during follow up (Figs 3C and 4C).

Thus, elevated values of BCN and CRP provided independent and incremental prognostic information. Likewise, risk classification analysis (Fig 4D) revealed also that the CRP reclassified 37.1% (p<0.027) and 51.4% (p<0.011) of patients when added to clinical model in combination with high BCN value. These results were confirmed by IDI analysis.

## Discussion

In our study, we evaluated the prognostic ability of four biomarkers (ST2, NT-proBNP, CRP and hs-cTnT) known to be involved in various pathophysiological pathways closely intertwined, in a population with stable chronic HF.

A combined multimarker model including ST2 and CRP identifies a best risk stratification for death for all-cause mortality as well as cardiovascular mortality. In addition, we confirm the prognostic value of Barcelona score and extend the previous study (10) showing an improvement in combination with CRP.

This study supports the use of ST2 measurements in a multimarker approach including ST2 and CRP, for assessing all cause death over 4 years in patients with chronic HF.

### Impact of ST2 as prognosis factor

HF is a complex disease involving various pathophysiological pathways reflected by numerous biomarkers and a biomarker alone, reflecting a single pathophysiological condition, cannot accurately account for all of these aspects [4]. Beyond conventional markers, ST2 has rapidly emerged as promising because of its pluripotent role in inflammation, mechanical strain, remodeling and fibrosis [10]. In addition, it offers advantages when compared to natriuretic peptides, since not affected by age, renal function or BMI [16]. A low intrapatient variation was reported by Wu et al. [17] (index of individuality<0.25) suggesting that ST2 may be more useful for monitoring long-term HF.

Despite its lack of specificity [18–21], ST2 appears consistently as promising in prognostic prediction of mortality, particularly in combination with natriuretic peptides among patients with chronic HF [10]. Here, we confirme that ST2 alone is an important risk factor for all cause or cardiovascular mortality in chronic HF patients. Even after adjustment for clinical variables including co-morbidities and several others biomarkers (including the gold-standard NT-proBNP), ST2 remains the strongest prognosis biomarker in our population. In addition to natriuretic peptides, ST2 could have additive value with other biomarker (hs-cTnT) or clinical parameters corroborating the interest of the Barcelona score in risk stratification, and providing an external validation.

### Relationship to CRP

Inflammation plays a key role in the progression of cardiac dysfunction [22]. As a result, high levels of CRP as well as other markers of inflammation such as TNF–alpha (Tumour Necrosis Factor-alpha) or GDF-15 (growth differentiation factor-15), have been shown to have significant prognostic and therapeutic implications in HF [3–7]. Our results are in line with these data, since we show a significant positive association between CRP levels and risk of all cause and cardiovascular disease mortality.

To the best of our knowledge, combined predictive power of biomarkers that reflect inflammation (CRP), abnormal left ventricular structure and dysfunction (NT-proBNP) and pluripotent effect on HF (ST2) has not been evaluated in previous studies. Our study provides evidence that the combination of high CRP (>6.4mg/L) and ST2 (>47.6ng/mL) dramatically increased the mortality risk and that the further association with NT-proBNP did not provide additive information. CRP, NT-proBNP and ST2 reflect distinct pathophysiological pathways involved in HF, so that their combination could identify synergistically more efficiently subgroups of patients at high risk of mortality.

In addition, the two biomarkers CRP and ST2 are easily accessible and reproducible, so that a multimarker model based on them should be easy to implement.

CRP is also correlated with Barcelona score [11] and in the model after adjustment by this score, CRP remains an independent predictive marker of mortality. It may be noted that in the Barcelona score [11], inflammation is perhaps not sufficiently taken into account, since the combination of the Barcelona score and CRP, provides additive information to improve the stratification. On the one hand, the presence of increased CRP and ST2, related in part to inflammation, confirms the existence of a chronically activated acute phase response in our HF population. On the other hand, adding the CRP to the score of Lupon et al. [11] (Fig 4), as well as adding CRP to ST2 (Fig 3), could improve the prediction of all cause and cardiovascular mortality. This hypothesis is strongly supported by the increase in the c-statistic index and by the Net Reclassification Index. These results, obtained in stable HF, are in total agreement with the recent report by Lassus et al. [23] suggesting the ability of a multimarker strategy using CRP and ST2 to predict mortality in acute HF [23]. Beyond their close correlation (rho = 0.47, p<0.001) (Fig 2), their combination further improves risk stratification allowing to discriminate well patients at low and high risk. As shown in Figs 3C and 4C, the mortality rate dramatically increases by a three to four-fold ratio when adding CRP to both ST2 or Barcelona score [11]. All these considerations taken in consideration suggest that we could use the combination of either CRP with ST2 or CRP with the Barcelona score. However, further studies are needed to confirm the interest of this approach in clinical practice, both for monitoring and therapy.

### Limitations

The study was limited by the unicentric design and the relatively small sample size. We only measured biomarkers at the time on the recruitment in the study, and only one blood sample was available. In consequence, we did not evaluate the monitoring of the biomarkers which can be also useful as monitoring markers. However, the low value of index of individuality for ST2 reported by Wu et al. [17] would indicate its role as a prognostic marker. In addition, until now, ST2 assay is not available in many centers, reducing the use of the multimarker strategy at present.

## Conclusion

ST2 may be considered as an additional marker of risk mortality in patients with HF, but the likely therapeutic benefit from lowering ST2 remains to be demonstrated in interventional studies. Because of its lack of specificity for cardiac stretch, ST2 should not be considered as a diagnostic marker but rather as a predictor of all cause and cardiovascular death. Our results extend previously published reports in which high ST2 levels are associated with an increased risk of death of all cause or cardiovascular mortality. Clearly, risk assessment has a significant impact on the management of patients with an adapted monitoring and aggressive therapy.

Overall, our findings extend previous data demonstrating that ST2 in combination with CRP as a valuable tool for identifying patients at risk of death. The multimarker approach could represent a promising tool combining markers involved in pathophysiology of HF.

## Supporting Information

S1 Fig Mean value of log ST2 according to the number of comorbidities from 0 to 6 in the study population of HF. (TIF)
