# Supplementary figures and images for: Multi-Marker Strategy in Heart Failure: Combination of ST2 and CRP Predicts Poor Outcome
Source: PLoS One. 2016 Jun 16;11(6):e0157159. doi: 10.1371/journal.pone.0157159 (PMC4911159; doi:10.1371/journal.pone.0157159)

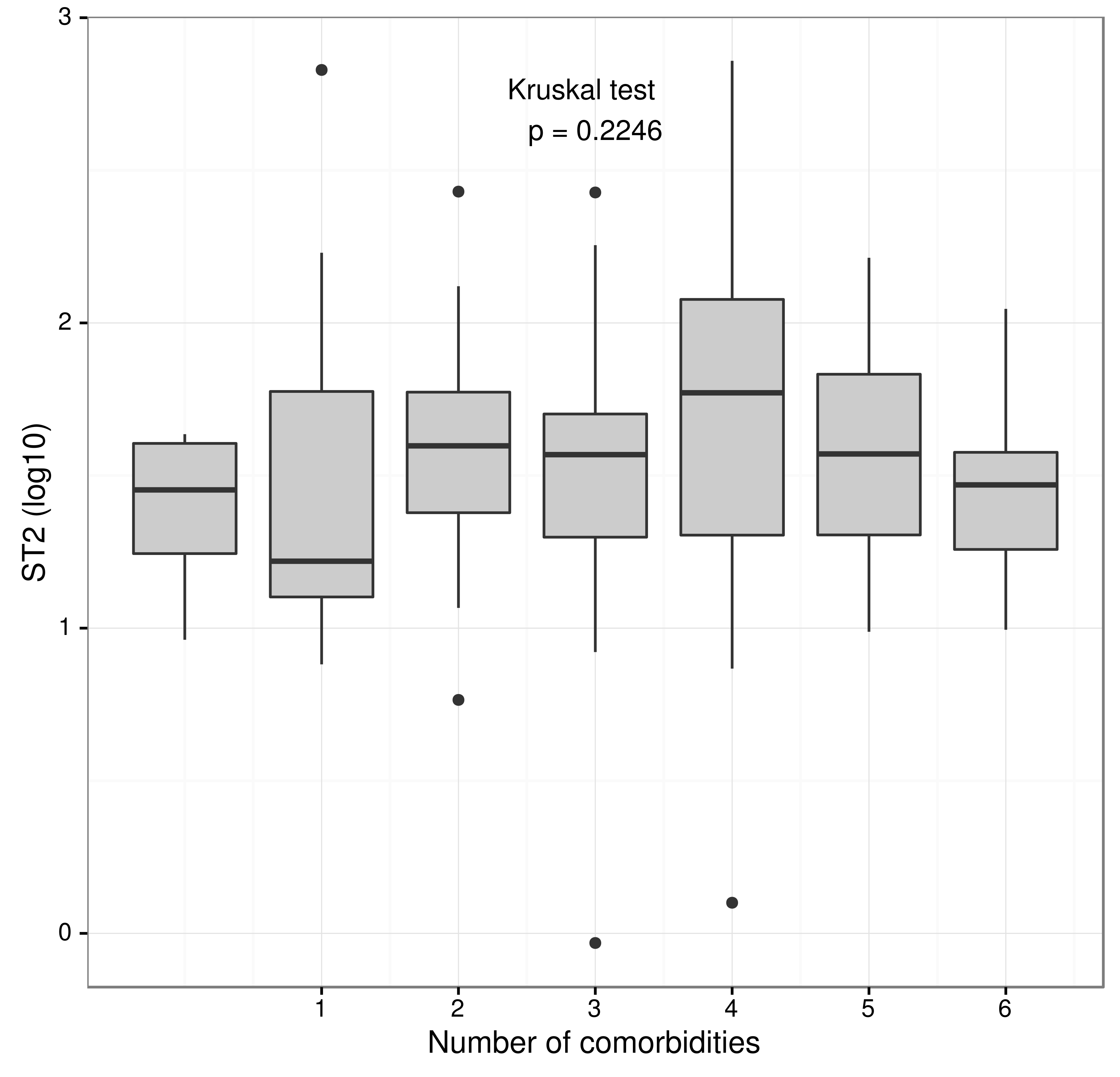

Supplement: S1 Fig — (TIF) [file pone.0157159.s001.TIF]
